# Defining the high-risk category of patients with cutaneous melanoma: a practical tool based on prognostic modeling

**DOI:** 10.3389/fmolb.2025.1543148

**Published:** 2025-02-07

**Authors:** Oleksandr Dudin, Ozar Mintser, Vitalii Gurianov, Nazarii Kobyliak, Denys Kozakov, Sofiia Livshun, Oksana Sulaieva

**Affiliations:** ^1^ Pathology Department, Medical Laboratory CSD, Kyiv, Ukraine; ^2^ Department of Fundamental Disciplines and Informatics, Shupyk National Healthcare University of Ukraine, Kyiv, Ukraine; ^3^ Endocrinology Department, Bogomolets National Medical University, Kyiv, Ukraine; ^4^ Kyiv Medical University, Pathology Department, Kyiv, Ukraine

**Keywords:** cutaneous melanoma, BRAF mutations, histological subtype, recurrence, prognostic model

## Abstract

**Introduction:**

Although most cutaneous melanoma (CM) in its early stages is treatable, the risk of recurrence remains high and there is a particular ambiguity on patients prognosis. This drives to identification of prognostic biomarkers for predicting CM recurrence to guide appropriate treatment in patients with localized melanoma.

**Aim:**

This study aimed to develop a prognostic model for assessing the risk of recurrence in patients with CM, enabling prompt prognosis-driven further clinical decision-making for high-risk patients.

**Materials and methods:**

This case-control study included 172 patients with CM recurrence (high-risk group) and 30 patients with stable remission (low-risk group) 3 years after primary diagnosis. The impact of sex, age at diagnosis, anatomical site, histological characteristics (the histological type, pathological stage, ulceration; the depth of invasion, mitotic rate, lymphovascular invasion, neurotropism, association with a nevus, tumor-infiltrating lymphocyte density, tumor regression and *BRAF* codon 600 mutation status) on CM recurrence was evaluated.

**Results:**

Five independent variables, including nodal status, a high mitotic rate, Breslow thickness, lymphovascular invasion, perineural invasion and regression features were identified as the most significant. A 5-factor logistic regression model was developed to assess the risk of melanoma recurrence. The sensitivity and specificity of the model were 86.1% and 72.7%, respectively.

**Conclusion:**

The developed model, which relies on routine histological features, allows the identification of individuals at high risk of CM recurrence to tailor their further management.

## Introduction

Cutaneous melanoma (CM) is a highly aggressive skin malignancy whose incidence has increased dramatically in recent decades (Siegel et al., 2022). Although CM represents approximately 4% of all skin malignancies, it is responsible for approximately 75% of skin cancer-related deaths (Gosman et al., 2023; Davis et al., 2019). According to the available data, a majority of melanoma cases diagnosed at stages III and IV are associated with high mortality (Dudin et al., 2023). More than 65%–90% of CMs diagnosed at early stages are treatable, with a high overall survival rate (Orzan et al., 2015). Nonetheless, the risk of recurrence remains high, reaching 50% in stage III melanoma (Garbe et al., 2022; Helvind et al., 2023). Even in localized stage I-II melanoma, 15%–20% of patients relapse locally or in/transit (Orzan et al., 2015), ∼50% in regional lymph nodes and ∼29% at distant metastatic sites (Salama et al., 2013). The follow-up strategy for CM patients depends on the stage of disease based on the criteria defined by the American Joint Committee on Cancer (AJCC) staging manual (8^th^ edition). The current guidelines, however, present a degree of ambiguity where the question of follow-up in patients with localized CM remains unresolved, with the recommendation to conduct only regular skin examinations during the first 5 years (Swetter et al., 2021). This drives researchers’ interest in identifying prognostic biomarkers for predicting CM recurrence and progression to guide appropriate treatment in patients with localized melanoma.

There are various patterns of recurrence, including local, satellite or transit metastases, as well as lymph node and systemic metastases. While advanced melanoma is prone to a high risk of systemic metastasis, early-stage CM tends to recur at the locoregional level (Peirano et al., 2023). The risk of CM recurrence is related to various factors, including stage, sex, age, depth of invasion, mitotic rate, host response to tumor growth, genetic alterations, etc*.* (Elder et al., 2005). The stage and melanoma thickness have been shown to play crucial roles in predicting tumor behavior and shaping patient management (Rothberg and Rimm, 2014). Sentinel lymph node (SLN) involvement in the development of malignant melanoma is associated with an increased risk of recurrence or progression (Lund, 2022). However, correct SLN identification and accurate assessment of lymph node status require advanced preoperative planning via 3D imaging with SPECT/CT for better intraoperative decision-making. Moreover, the rate of false-negative results in SLN assessment is still high, ranging from 5% to 21% (Peirano et al., 2023).

Similarly, Breslow thickness, ulceration, the rate of proliferation, the anatomical site, and the CM histological subtype have also been reported to be prognostically significant factors for predicting tumor behavior and disease progression. The recent systematic review devoted to identifying prognostic models for melanoma survival, recurrence and metastasis in patients with CM of I and II stages highlighted the feasibility of using clinicopathological features for prognostication. The most common features used for prognostic models included ulceration, Breslow thickness/depth, sociodemographic status and primary site of melanoma lesions (Kunonga et al., 2023). Other studies confirmed the prognostic significance of CM location and ulceration (Rashid et al., 2011). They also illuminated the role of histological type and patients age. Besides tumor-infiltrating lymphocytes (TILs) were shown to have prognostic and predictive value in some types of CM (Lee et al., 2013). While Wan et al. (2022) applied machine-learning algorithms for predicting CM recurrence based on 36 clinical and histopathologic features and demonstrated that Breslow tumor thickness and mitotic rate were the most predictive features (Wan et al., 2022). Nevertheless, the accuracy and affordability of various prognostic models based on clinical and histological parameters is still under debate. Assessment of these parameters might not be sufficient to identify individuals at high risk of recurrence (Salama et al., 2013; Homsi et al., 2005). Various immunohistochemical markers, including Bcl-6, MUC18, metalloproteinase-2, Ki-67, p16, p27, iNOS, etc. (Alonso et al., 2004; Ekmekcioglu et al., 2006), are related to melanoma prognosis, yet only a few of these markers have been confirmed to be linked to the likelihood of recurrence in CM patients (Ding et al., 2022). Several novel biomarkers, including exosomal melanoma inhibitory activity (MIA), serum S100B, epidermal AMBRA1 and loricrin, have been described in the context of disease prognosis. For example, the loss of peritumoral AMBRA1 and loricrin has been considered a prognostic biomarker of a low risk of recurrence in patients with stage I-II melanoma (Ewen et al., 2023). In the context of immunotherapy, the biomarkers LAG3 and TIGIT and tumor-infiltrating immune cell signatures have been described as both prognostic and predictive in CM (Naimy et al., 2023). Similarly, the expression profiles of ferroptosis genes together with clinical data from The Cancer Genome Atlas (TCGA) database were used to construct a model for predicting disease progression. The model stratified patients into low-risk and high-risk groups according to the prognostic value of ferroptosis-related gene expression. The expression profiles of ferroptosis-related genes correlate with disease progression in patients with melanoma. However, immune-activating pathway expression was related to the low-risk group (Kunonga et al., 2023). Although the stratification of patients is effective for evaluating disease progression, the limited data and availability of these biomarkers for routine testing prevent their application in clinical practice (Ding et al., 2022). The novel prognostic model can effectively stratify patients with respect to disease-associated risk; however, the currently available data to support these models to aid in clinical decision-making are still lacking (Wen et al., 2020). Risk factor-driven models and risk-associated biomarkers of CM recurrence are yet to provide commonly available practical tools for stratifying high-risk groups of patients and guiding further decision-making.

This study aims to develop a prognostic model for assessing the risk of recurrence in patients with CM, enabling prompt prognosis-driven further clinical decision-making for high-risk patients.

## Materials and methods

### Setting and participants

A total of 202 CM patients were included in this case-control study. The patients enrolled in this study had a history of observation for at least 3 years within the period from 2017 to 2022. This study was submitted for and formally exempted from Institutional Review Board approval because of the anonymous nature of the retrieved retrospective data. Informed consent was waived because of the fully anonymous nature of the delivery of the retrospective study data.

### Methodology

Clinical, histopathological and molecular testing on BRAF mutation status data were retrieved from the database. In the first step, we selected all cases with histologically confirmed CM with complete clinical, histological and molecular data. Next, we selected only cases obtained by excision with histologically confirmed negative surgical (resection) margins. Finally, only cases with follow-up with histological data available were enrolled in the study cohort. The following inclusion criteria were applied: histologically confirmed diagnosis of CM of stages I-III, negative surgical (resection) margins after excision (to exclude the direct impact of positive margins on melanoma recurrence), known pathological stage and histological tumor features, and known *BRAF* codon 600 mutation status, follow-up histological data within 3 years after diagnosis. The exclusion criteria were as follows: incisional biopsy of skin melanoma instead of excision, positive surgical (resection) margins, lack of histological data according to the CAP protocol, presence of distant metastasis at the time of primary diagnosis, unknown *BRAF* status, and lack of follow-up data with confirmed outcomes. Thus, all patients were characterized with respect to clinical and histopathological tumor features and were tested for *BRAF* codon 600 mutations.

To estimate the sample size, we used the G*Power statistical power analysis tool and calculated sample size for α = 0.05, Power = 0.8 and strong influence of the factor (OR ≤ 0.33) (Faul et al., 2007). According to calculations the minimal sample size was equal to 152 patients.

According to the results of follow-up histology and defined outcome patients we divided into groups. The high-risk group included 172 patients with melanoma recurrence within 3 years after primary diagnosis. Recurrence status was recorded in cases of true scar recurrence, local satellite/in-transit recurrence, and nodal or distant metastasis. Thirty patients who achieved stable remission 3 years after primary diagnosis were assigned to the low-risk group.

### Methods and variables analyzed

The collected clinicopathological data included the patient’s sex, age at diagnosis, and anatomical site of the primary melanoma. Relevant histological characteristics according to CAP protocols for CM were retrieved. The data included the histological type of CM according to the WHO classification (WHO); pathological stage, including tumor size (pT) and lymph node status (pN); ulceration; and tumor regression. The depth of invasion was evaluated according to the maximum tumor (Breslow) thickness (in mm) and anatomic (Clark) level. Breslow thickness (or maximum tumor thickness) was measured with an ocular micrometer at a right angle to the lesion surface from the upper edge of the granular layer of the epidermis (or the base of the ulcer in case of ulceration) to the deepest site of tumor invasion. Foci of neurotropism, lymphovascular invasion or microsatellites were not included in tumor thickness measurements. Anatomic (Clark) levels were identified according to CAP protocol as follows: I - Intraepidermal tumor growth (melanoma *in situ*), II - Tumor present in but does not fill and/or expand papillary dermis, III - Tumor fills and expands papillary dermis, IV - Tumor invades into reticular dermis, V Tumor invades subcutaneous layer.

The roles of factors such as the mitotic rate (per 1 mm^2^), lymphovascular invasion, neurotropism (perineural or intraneural invasion), association with a nevus, tumor-infiltrating lymphocyte density, and tumor regression at the time of primary diagnosis were assessed in terms of patient prognosis. Tumor regression was defined by the following features: replacement of tumor cells by lymphohistiocytic infiltration, or attenuation of the epidermis and non-laminated dermal fibrosis with inflammatory cells, melanophagocytosis, and telangiectasia (Aung et al., 2017).

In addition, the potential impact of the *BRAF* codon 600 mutation status on CM recurrence was evaluated. Tumor-infiltrating lymphocytes (TILs) were evaluated in a dichotomous manner according to the pathology report description: the absence of lymphocytes or a low number of tumor-infiltrated lymphocytes were considered TIL-low infiltration, whereas moderate or high-intensity lymphocytic infiltrates were considered TIL-high infiltration.

Molecular testing for detecting BRAF codon 600 mutations was conducted on tissue samples via formalin-fixed paraffin-embedded blocks with verified tumor content. Ten 10 μm-thick sections were obtained from each paraffin block containing a representative tumor area (>20% tumor cells, >200 cells in the sample, <20% necrosis area). DNA was extracted using ZYTOVISION VisionArray FFPE DNA Extraction Kit according to the manufacturer’s instructions. The detection of BRAF codon 600 mutations was performed by real-time polymerase chain reaction (RT‒PCR) using Easy PGX-ready BRAF system (Diatech Pharmacogenetics, Italy). The assay is designed to detect 5 types of *BRAF* mutations in codon 600: V600E (1799T > A), V600E (1799_1800TG > AA), V600 K (1798_1799GT > AA), V600D (1799_1800TG > AT), and V600 R (1798_1799GT > AG).

### Statistical analysis

Statistical analysis was conducted using MedCalc® Statistical Software version 22.016 (MedCalc Software Ltd., Ostend, Belgium; https://www.medcalc.org; 2023) and GraphPad Prism (GraphPad Prism Version 10.0.3 (217) GraphPad Software, San Diego, California, United States; www.graphpad.com). Descriptive statistics for continuous variables (such as age, mitotic rate, and Breslow thickness) were presented as the Mean and SEM for normally distributed data or Median and Interquartile Range for non-normally distributed data. Quantitative data were assessed as frequencies (%). The χ^2^ test or Fisher’s exact test were applied to compare frequencies. An unpaired *t*-test or non-parametric Mann-Whitney test were used to compare continuous variables between high- and low-risk groups. The developed model was developed to predict the risk of melanoma recurrence within 3 years (binary outcome), so logistic regression was used. Univariate and multivariate logistic regression analysis models were used to assess the impact of various variables on the risk of relapse. The stepwise method was used to identify the set of variables with the highest impact on outcome and define the best-fitting multivariable logistic regression model. To assess the effect of variables on the outcome, odds ratios (OR) with 95% confidence interval (95% CI) were calculated. The diagnostic performance of the logistic regression models was evaluated using Receiver Operating Characteristic (ROC) curve analysis. The area under the ROC curve (AUROC) and its 95% CI were calculated. The P-value <0.05 was considered statistically significant for all of the tests.

## Results

### Patients’ characteristics

A total of 202 cases with primary CM and 3-year follow-up data are reported in this study. Among the enrolled cases, 103 were males (51%) and 99 (49%) were females, aged 52.6 ± 1.51 (95% CI 49.6–55.6) and 52.0 ± 1.46 (95% CI 49.2–54.9), respectively. The high-risk group included 84 males (48.9%) and 88 females (51.1%). Among patients with remission, there was a higher rate of males (19 of 30, 63.3%) and females represented 36.7% (11 of 30) of the group.

The high-risk group included 172 patients aged 52.2 ± 1.14 years (95% CI 49.9–54.5). The low-risk group comprised 30 patients with stable remission aged 52.9 ± 2.76 years (95% CI 47.3–58.54). There was no significant difference in age and gender distribution between the high- and low-risk groups (Table 1).

**TABLE 1:** Characteristics of patients of the study.

Parameters	Total (n = 202)	High-risk group (recurrence) (n = 172)	Low-risk group (remission) (n = 30)	P-value
Age, years	52.3 + 1.05 (50.3–54.4)	52.2 ± 1.14 (49.9–54.5)	52.9 ± 2.76 (47.3–58.54)	0.819
Sex				
Male	103 (51.0%)	84 (48.9%)	19 (63.3%)	0.618
Female	99 (49.0%)	88 (51.1%)	11 (36.7%)	
The stage at the time of diagnosis				
I	19 (9.4%)	13 (7.6%)	6 (20%)	<0.001
II	133 (65.8%)	109 (63.4%)	24 (80%)	
III	50 (24.8%)	50 (29.0%)	0	
Anatomical site of the primary tumor				
Face & Scalp	17 (8.4%)	16 (9.3%)	1 (3.3%)	0.645
Limbs	56 (26.7%)	47 (27.3%)	9 (30%)	
Trunk	75 (37.1%0	62 (36.1%)	13 (43.3%)	
NOS	54 (26.7%)	47 (27.3%)	7 (23.4%)	
Histological type				
SSM	93 (46%)	77 (44.8%)	16 (53.3%)	0.518
NM	56 (27.7%)	47 (27.3%)	9 (30%)	
Spitzoid + Desmoplastic	7 (3.5%)	7 (4.1%)	0	
NOS	46 (22.8%)	41 (23.8%)	5 (16.7%)	
BRAF codon 600 mutation status				
*BRAF*-mutated	124 (61.4%)	106 (61.6%)	18 (60%)	>0.999
*BRAF-*wt	78 (39.6%)	66 (39.4%)	12 (40%)	

Data presented as M±SE (95%CI) or % (n).

In both groups CM at trunk predominated comprising 36.1% in high-risk group and 43.3% in low-risk patients. Similarly, there were no differences in the frequency of various histological types of CM between groups. The most common types were SSM (44.8% and 53.5% of cases in high- and low-risk groups, respectively) and NM comprising correspondingly 27.3% and 30%. At the same time, 7 cases (4.1%) were reported with Spitzoid or desmoplastic CM in the high-risk group. However, we did not find statistically significant differences in the anatomical site of the primary tumor between groups.

Among the observed cases, 19 (9.4%) were characterized as stage I, 133 (65.8%) as stage II and 50 (24.8%) as stage III CM. All patients with affected lymph nodes (stage III) were in the high-risk group, with confirmed recurrence. CM recurrence was characterized by either locoregional or distant metastasis within 3 years of the follow-up period. Within the high-risk group, 13 cases (7.6%) were identified as stage I, 109 cases (63.4%) as stage II, and 50 cases (29%) as stage III CM according to the AJCC tumor staging system. Alternatively, the low-risk group demonstrated 20% of Stage I and 80% of Stage II CM. There were no node-positive cases in this group. This defined a statistically significant difference in staging between patients with recurrence and remission (P < o.oo1).

Finally, we did not find a difference in the BRAF mutation rate between groups, which reached 61.6% in the high-risk group and 60% in patients with remission.

### The impact of histological and molecular features on CM recurrence

Regarding the anatomical site of the primary tumor, most cases (n = 75, 37.1%) were located at the trunk, 56 CMs (26.7%) were located in the upper or lower limbs, 17 at the face or scalp areas (8.4%), and the remaining 54 cases had no specified primary CM site (26.7%). There was no significant difference between the groups in the anatomical site of the primary tumor (P = 0.645), the histological subtype of CM (P = 0.518), or the incidence of *BRAF* codon 600 mutation (P > 0.999).

**FIGURE 1:**
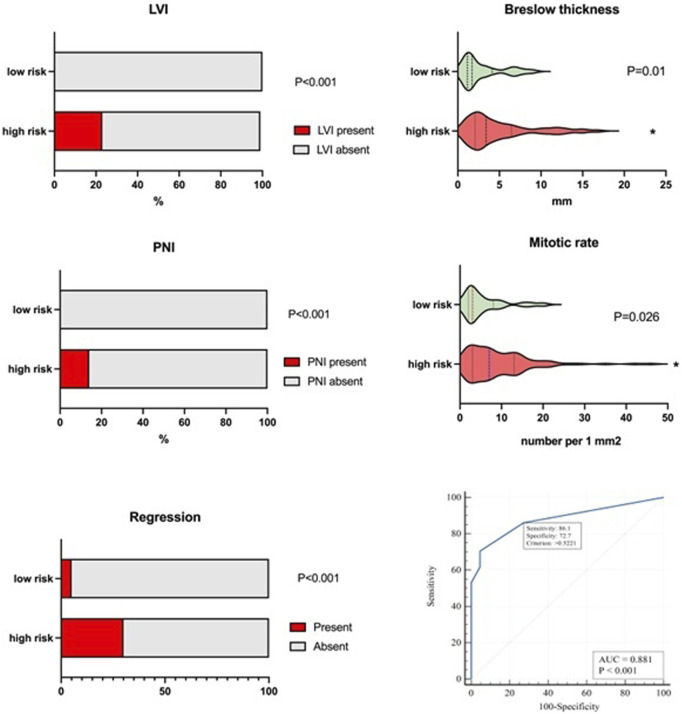
Difference in histological features between high- and low-risk groups. The high-risk group demonstrated a significantly higher rate of mitosis, depth of invasion assessed via Breslow thickness, LVI and PNI, and tumor regression features. ROC curve of these 5-factorial logistic regression model for predicting the risk of CM recurrence demonstrates the area under the curve of AUC = 0.88 (95% CІ 0.81–0.93) reflecting the strong link between the risk of recurrence and the selected variables The sensitivity and specificity of the model are 86.1% (95% CІ 78.4%–91.8%) and 72.7% (95% CІ 49.8%–89.3%), respectively.

Although there was no difference in ulceration of primary melanoma between high- and low-risk groups (P = 0.241), we observed a significantly greater mitotic rate (P = 0.026) and Breslow thickness (P = 0.01) in the high-risk group than in the low-risk group. At the same time, LVI (P < 0.001), PNI (P < 0.001) and tumor regression features (P < 0.001) were observed at a higher rate in patients with CM recurrence (Figure 1).

There were 124 *BRAF-*mutation-positive cases (61.4% of the observed cohort). The rate of BRAF codon 600 mutations did not differ between the high- and low-risk groups (Table 2). In patients with recurrence, BRAF codon 600 mutation was detected in 61.6% of patients (106 of 172), whereas in the low-risk group, it was identified in 60% of cases (17 of 30, P > 0.999).

**TABLE 2:** Impact of clinical and histopathological features on the risk of recurrence in stage I-III CM (in univariable logistic regression model).

Variables		Model coefficient, b ± m	P	Or (95% CI)	AUROC (95% CI)
Sex	F	Referent			–
	M	−0.59 + 0.41	0.147	–	
Stage		1.49 ± 0.41	<0.001	4.42 (1.98–9.87)	0.69 (0.62–0.75)
Age		−0.003 + 0.013	0.519	–	–
Anatomical site of primary tumor	NOS	Referent			–
	Limbs	−0.25 ± 0.54	0.644	–	
	Face or Scapl	0.87 ± 1.11	0.433	–	
	Trunk	−0.34 ± 0.51	0.500	–	
BRAF wt Vs. mutation		0.07 ± 0.40	0.866	–	–
Mutation type	WT	Referent			–
	V600 K	0.78 ± 1.09	0.473	–	
	V600 E	0.01 ± 0.41	0.990	–	
Positive Nodal status (Stage 3)		1.49 ± 0.41	<0.001	4.41 (1.98–9.87)	0.69 (0.62–0.75)
Histological type	NOS	Referent			0.60 (0.53–0.66)
	NM	−0.73 ± 0.59	0.216	–	
	Spitz	18.1	0.764	–	
	SSM	−0.86 ± 0.54	0.120	–	
Ulceration		0.32 ± 0.45	0.482	–	–
Mitosis more than 5		0.099 ± 0.045	0.028	1.10 (1.01–1.21)	0.66 (0.57–0.73)
Clark level		0.50 ± 0.27	0.063	–	–
Breslow thickness		0.29 ± 0.11	0.007	1.33 (1.08–1.64)	0.71 (0.63–0.79)
TILs high		−0.15 ± 0.21	0.478	–	–
LVI presence		21.1	0.031	22.2 (1.32–373)	0.64 (0.56–0.72)
PNI presence		19.9	0.138	–	0.57 (0.49–0.65)
Regression presence		2.25 ± 1.04	0.031	9.51 (1.23–73.3)	0.63 (0.54–0.71)
Microsatellite presence		1.16 ± 1.06	0.274	–	–
Association with nevus		1.33 ± 1.06	0.207	–	–

Nodal status, BRAF, status, ulceration, LVI, PNI, regression and microsatellite were considered as binary variables. The presence of the feature was considered when assessing the model coefficients.

### Logistic regression analysis for predicting CM recurrence

Single-factorial logistic regression analysis was conducted to identify the features with a moderate degree of relationship with patient outcome (AUROC = 0.6–0.7). These factors included the following: nodal status (stage), mitotic rate, Breslow thickness, LVI, PNI, and regression features.

The risk of recurrence is increased significantly in N-positive tumors (stage III) (P < 0.001; OR = 4.41; 95% CІ 1.98–9.87). Similarly, a high mitotic rate, Breslow thickness, LVI, PNI, and regression features affected the probability of CM recurrence (Table 2). To define the minimal set of variables for predicting CM recurrence in a multifactorial logistic regression model, stepwise analysis was employed (stepwise threshold of inclusion p < 0.05, threshold of exclusion p > 0.1). Five independent variables were identified as the most significant. The independent variables were independent of each other (the Variance Inflation Factor for predictors did not exceed 1.1). Clark level, Breslow thickness correlated with the Stage (r = 0.47 and r = 0.45, p < 0.001, correspondingly), they were excluded from the multiple regression model using Stepwise method. The 5-factors regression model includes only independent significant variables. If we add Breslow’s thickness to the model (for example) then the coefficient does not differ to zero (P = 0.531).

The adequacy of the constructed model was confirmed by its characteristics (χ^2^ = 42.9, at 5 degrees of freedom; P < 0.001). The AUROC of 0.88 (95% CI 0.81–0.93) reflects the strong link between the risk of progression and tumor characteristics such as stage, mitotic rate, LVI, PNI and tumor regression features (Table 3; Figure 1). When defining the optimal model threshold Y_crit_ > 0.5221 the sensitivity and specificity of the model were 86.1% (95% CІ 78.4%–91.8%) and 72.7% (95% CІ 49.8%–89.3%), respectively. The positive predictive value PPV comprised 94.3% (95% CІ 89.3%–97.0%), while the negative predictive value NPV was found to reach 50% (95% CІ 37.2%–2.8%). The model can be represented by Formula 1:

$$\ln\left(Y/\left(1-Y\right)\right)\;=21.6*X1+1.02*X2+22.1*X3+21.6*X4+2.49*X5+0.09$$ (1)

where Y is the risk of CM recurrence; X1 = 0 for Stages 1–2 and X1 = 1 for Stage 3; X2 = 0 for mitotic rate <=5 and X2 = 1 for mitotic rate>5; X3 = LVI (0/1); X3 = PNI (0/1); and X5 = regression (0/1).

**TABLE 3:** Characteristics of the 5-variables logistic regression model for assessing the risk of CM recurrence.

Variables		Model coefficient, b ± m	P	Or (95% CІ)	AUROC (95% CІ)
Stage	1–2	Referent			0.88 (0.81–0.93)
	3	21.8	All patients had a recurrence		
Mitotic rate	≤5	Referent			
	>5	1.02 ± 0.58	0.078	4.57 (1.26–16.6)	
LVI	0	Referent			
	1	22.1	All patients had a recurrence		
PNI	0	Referent			
	1	21.6	All patients had a recurrence		
Regression		2.49 ± 1.07	0.020	12.0 (1.48–97.9)	

For practical application of the 5-factor model, the tool for calculating patient risk prediction was implemented in Excel.

## Discussion

Although various indicators are considered prognostic at the time of diagnosis, predicting the risk of recurrence in early-stage CM is still challenging. Whereas it is widely accepted that the clinicopathological and demographic features of primary tumors impact prognosis, various authors have applied different sets of histopathological criteria to predict melanoma recurrence (Abbas et al., 2014; Chousakos et al., 2023). Here, we evaluated the relationships between demographic, clinical, and histopathological data and patient outcomes. We analyzed the effects of positive nodal status, histological type, mitotic rate, Breslow thickness, LVI, PNI and tumor regression features on the risk of CM recurrence. Moreover, we found no impact of age, sex, Clark level of invasion, microsatellites, ulceration, association with nevus, or the presence or type of *BRAF* codon 600 mutations on patient prognosis. Ulceration, Breslow thickness and the mitotic rate were found to have the highest statistical power for predicting outcomes in a study by Vita et al. (2023). Besides, multivariable analysis by Buja A. et al. revealed that age, primary tumor site, histological subtype, mitotic count, and tumor stage were independently associated with disease prognosis (Buja et al., 2021). The differences in prognostic criteria could be explained by the heterogeneity of CM patient populations used in these studies, various inclusion and exclusion criteria, and different endpoints. Importantly, the lists of selected primary factors to be analyzed for predicting patient outcomes presented a high degree of variability between the studies. The common idea, however, is based upon the need to identify subgroups of CM patients at a higher risk for recurrence to optimize management for such patients (Chousakos et al., 2023).

This study elucidated the 5 main tumor features associated with a high risk of CM recurrence, including positive nodal status (stage III), mitotic rate, lymphovascular or perineural invasion, and features of tumor regression. TNM staging was previously shown to be vital in predicting melanoma outcomes (Cozzolino et al., 2023). This enables to prognose the patient outcome based on five widely used features reported by pathologists according to standard protocols. The simplicity and affordability of variables used for assessing the risk of recurrence make the developed model attractive for clinical application at least for the preliminary risk assessment and making decisions concerning every particular patient management based on his or her stage, mitotic rate, tumor invasiveness and regression features.

Comparing the developed model with already existing tools it is important to highlight that many studies apply machine learning-based algorithms which also defined the significance of variables in this study. For instance, using artificial intelligence tools for predicting short-term mortality in CM patients, Aung et al. (2017) showed that both distant and nodal metastasis aggravated the outcome in patients with CM. In addition, patient age, sex, tumor site, histological type and growth phase also contributed significantly to predicting overall survival. In our study focused on predicting CM recurrence, we also demonstrated the role of staging and underscored the prognostic role of other factors. Notably, the mitotic rate and tumor invasiveness features were considered to be tightly linked to melanoma aggressiveness and prognosis in recent studies (Thompson et al., 2011). Another study utilizing such tools as MLP, Adaptive Boosting (AB), Bagging (BAG), logistic regression (LR), Gradient Boosting Machine (GBM), and eXtreme Gradient Boosting (XGB) algorithms developed a model for predicting metastasis in patients with nodular melanoma (Serra et al., 2024). The MLP was found as the optimal. It demonstrated the best parameters reaching AUC = 0.932, F1 = 0.855, Accuracy = 0.856, Sensitivity = 0.878. In contrast to our study, this model was focused on nodular melanoma. However, it also highlighted the prognostic significance of the primary site and stage. Another cohort study performed at 4,718 patients with CM developed a model for prognosticating brain metastasis. Based on multivariate logistic regression analysis, authors identified the following significant risk factors of CNS metastasis of melanoma: a higher Breslow index, mitotic rate ≥1 mm^2^, ulceration, and microscopic satellites. These data partly correlate with our findings, although it included patients with stage IV, mucosal melanoma and was focused on CNS metastasis and outcome (Serra et al., 2024). Similarly, in Romanian study, the Breslow thickness >2 mm, high Clark level, high mitotic rate and ulceration were defined as the most significant prognostic factors for lymph nodal involvement in CM (Vița et al., 2023). Other studies also illuminated the prognostic significance of LVI associated with recurrent disease (P = 0.003) and metastatic disease (P = 0.008) (Tas and Erturk, 2017). Multivariate analysis also uncovered that lymph node metastasis, Breslow thickness, LVI, and angiotropism are predictors of the overall survival of patients with CM. Moreover, LVI was also shown to correlate with neurotropism, Breslow’s thickness and lymph node involvement (Tas and Erturk, 2017). Alternatively in an early-stage melanoma study performed on 1,720 patients, utilizing machine learning algorithms, only Breslow tumor thickness and mitotic rate were identified as the most informative features. Notably, models were evaluated internally by five-fold cross-validation of the MGB cohort, and externally via independent evaluation of training and testing cohorts. A recurrence classification performance of AUC in the internal and external validations comprised 0.845 and 0.812, respectively (Wan et al., 2022). These results are comparable with the performance of the model developed in our study.

Although Breslow thickness is an important histological parameter that can predict the outcome of primary CMs, this factor had a less prominent effect on the risk of recurrence in the observed cohort than the other factors selected for this model. According to the National Comprehensive Cancer Network guidelines, SLN biopsy is recommended for all CMs thicker than 1 mm (Carr et al., 2022). Nevertheless, some patients with thin lesions develop local or distant metastasis. This provoked a discussion on whether sentinel lymphadenectomy should be performed in addition to wide local excision for primary lesions ≤1.00 mm. Several studies have evaluated different approaches for identifying histopathological features and/or genetic markers for predicting SLN positivity and improving patient management (Carr et al., 2022). Although recommendations for SLN biopsy and complete lymph node dissection are still under discussion, the correct assessment of lymph node status is essential for accurate CM staging and prognosis, as it allows stratification of low- and high-risk groups and leads to improvements in regional disease control (Faries et al., 2017). Nevertheless, further prospective studies and clinical validation of the developed models are needed to implement personalized risk-assessment and management.

Interestingly, regression in primary CM was defined in our study as an important prognostic factor. According to recent studies, the prognostic value of regression in CM is quite controversial. While some studies have demonstrated a lack of correlation between regression and patient outcomes (Kaur et al., 2008), other authors have reported that regression predicts a greater risk of lymph node involvement (Oláh et al., 2003) and CM metastasis (Guitart et al., 2002). These controversies may be related to discrepancies in regression definitions and assessments, the different stages of melanoma included in the respective studies, the subjectivity of regression reporting and interpretation, the treatment used, *etc.* (McClain et al., 2012). Histologically, regression is characterized by a decrease in the number of melanoma cells associated with the host response, including inflammatory infiltrates, dermal fibrosis, melanophages, an increased number of ectatic blood and lymphatic vessels, and the apoptosis of keratinocytes or melanocytes (Aung et al., 2017). It reflects the tumor-host interplay at the site of the primary disease (Aung et al., 2017).

Tumor-host interplay in areas of regression can affect CM outcomes in different ways via changes in the tumor immune microenvironment (TIME), the selection of tumor subclones with different genetic profiles or the use of various cell death mechanisms. First, the melanoma-host interplay can significantly affect various phases of tumor growth and development, depending on the host immune response. This may involve various populations of lymphocytes and macrophages and be associated with alternative polarization of the immune reaction. Importantly, a recent study revealed that regression is highly correlated with TILs (Morrison et al., 2022). Importantly, however, only TILs, but not regression features, were linked with SLN status and survival in CM. Thus, the presence of CD8^+^ tumor-infiltrating lymphocytes impacts patient outcomes of CM. Alternatively, Yun et al. suggested that the negative impact of regression on CM prognosis can be related to increased dermal lymphatic vessel density, resulting in an enhanced risk of lymphovascular invasion (Yun et al., 2011). Moreover, the elimination of recognizable subpopulations of melanoma cells can benefit the remaining clones of aggressive melanoma cells, assisting their growth and tumor development.

Importantly, the predictive strength of the tumor regression features in our model was lower than that of stage or melanoma invasiveness. It works as a predictor of higher-risk CM only when it is combined with other factors, such as increased mitotic activity or perineural invasion, indicating aggressive melanoma tumor behavior. Importantly, the elimination of melanoma cells in areas of regression can be realized by various immune cells and through different cell death mechanisms. Aside from the classic antitumor immune reaction through CD8^+^ cytotoxic lymphocytes, alternative activation of the host immune response, with the prevalence of Ms-macrophages, myeloid-derived immunosuppressive cells or activation of immune escape mechanisms, can foster and shape the further development of malignancies (Mashukov et al., 2021; Stakhovskyi et al., 2022). Within their works on deciphering TIME-related expression, Liang Z. and coauthors revealed several promising immune-related biomarkers, demonstrating that high expression levels of the *GZMB*, *C1QA*, and *C1QB* genes correlate with favorable prognosis in patients (Liang et al., 2022). Similarly, Zhang et al. demonstrated the relevance of TIME assessment via genomic and epigenomic scores for defining high-risk and low-risk groups of CM patients to guide personalized melanoma treatment (Zhang et al., 2023). Finally, the tumor regression features in primary CM may be related to the shift between the apoptosis and necroptosis pathways (Yang et al., 2023). A lack of apoptosis signaling can lead to an alternative cell death pathway known as necroptosis, a recently discovered pathway of programmed cell death that bypasses apoptosis and might be involved in pathological oncogenic processes. This process is associated with mitochondrial dysfunction and is promoted by increased generation of reactive oxygen species in melanoma cells, resulting in mutagenesis and cell death (Basit et al., 2017). Recently developed models for prognosis and accurate prediction of the response to immunotherapy demonstrated a close link between necroptosis-related genes and immune cell signatures, identifying novel approaches for identifying high-risk patients and personalizing their treatment.

Heterogeneity of the existing data and models concerning the risk factors and prognostic models allowing to prediction of CM recurrence are related to numerous factors, including differences in populations involved and CM characteristics (stage, histological types, etc.), clinical and pathological data sets, approaches for data handling and outcomes applied. This reflects insufficient evidence to make robust conclusions for clinical decision-making in the management of patients with CM. The developed model could provide clinical usefulness for stratifying patients with a high risk of melanoma recurrence. Despite the relatively low figures of NPV, the model enables predicting the high risk of recurrence with good accuracy. Considering existing clinical data concerning the risk of CM recurrence varying according to different data from 50% to 80% in CM of I-III stages, the developed model allows for prediction recurrence in 94.1% of patients with CM of I-III stage though further external and clinical validation of the model are needed (Leiter et al., 2014; Stucky et al., 2010). In the future, a prognostic model could be used to tailor patient management during counseling and/or integrate predictive models in electronic healthcare systems. Accurate risk assessment could support physicians’ and patients’ decision-making in clinical settings to plan individualized follow-up and treatment to prevent disease progression and improve patients’ outcomes. The developed model could be also adjusted for navigating patients’ management after immunotherapy. However future multi-center trials are needed to justify the model’s application in populations treated with immunotherapy. The findings of the study also stimulate a comprehensive investigation of tumor regression mechanisms and interpretations for a better understanding of its role in CM behavior and progression.

## Conclusion

This study demonstrated the prognostic significance of tumor stage, mitosis rate, and invasion features, as well as tumor regression features, on CM patient outcomes. The application of the 5-factor model, which is based on routinely assessed histological markers, allows the definition of high-risk groups of patients with a high likelihood of CM recurrence. Its application could be useful for guiding guide personalized management strategies. Further prospective studies are needed to validate the model.

### Limitations of the study

This study is limited to a retrospective analysis of patient data over a three-year follow-up period. This study did not focus on the therapeutic schemes used for various patients in the cohort. Moreover, the sample included only patients with CM of the I-III stage, and we did not differentiate outcomes between groups. Due to the retrospective type of the study, there is a potential bias in data collection. For instance, the high- and low-risk groups were not equal in terms of CM staging. Logistic regression analysis instead of Cox proportional hazards regression was applied because of the lack of accurate follow-up data concerning the timing of recurrence onset. The developed prognostic model was based on logistic regression analysis and needs validation in further multicenter studies.

## Data Availability

The raw data supporting the conclusions of this article will be made available by the authors, without undue reservation.

## References

[B1] Abbas O. Miller D. D. Bhawan J. (2014). Cutaneous malignant melanoma: update on diagnostic and prognostic biomarkers. Am. J. Dermatopathol. 36, 363–379. 10.1097/DAD.0B013E31828A2EC5 24803061

[B2] Alonso S. R. Ortiz P. Pollán M. Pérez-Gómez B. Sánchez L. Acuña M. J. (2004). Progression in cutaneous malignant melanoma is associated with distinct expression profiles: a tissue microarray-based study. Am. J. pathology 164, 193–203. 10.1016/S0002-9440(10)63110-0 PMC1602212 14695333

[B3] Aung P. P. Nagarajan P. Prieto V. G. (2017). Regression in primary cutaneous melanoma: etiopathogenesis and clinical significance. Laboratory investigation; a J. Tech. methods pathology 97, 657–668. 10.1038/LABINVEST.2017.8 28240749

[B4] Basit F. Van Oppen LMPE Schöckel L. Bossenbroek H. M. Van Emst-De Vries S. E. Hermeling J. C. W. (2017). Mitochondrial complex I inhibition triggers a mitophagy-dependent ROS increase leading to necroptosis and ferroptosis in melanoma cells. Cell death and Dis. 8, e2716. 10.1038/CDDIS.2017.133 PMC5386536 28358377

[B5] Buja A. Bardin A. Damiani G. Zorzi M. De Toni C. Fusinato R. (2021). Prognosis for cutaneous melanoma by clinical and pathological profile: a population-based study. Front. Oncol. 11, 737399. 10.3389/FONC.2021.737399 34868928 PMC8634953

[B6] Carr M. J. Monzon F. A. Zager J. S. (2022). Sentinel lymph node biopsy in melanoma: beyond histologic factors. Clin. and Exp. metastasis 39, 29–38. 10.1007/S10585-021-10089-9 34100196

[B7] Chousakos E. Zugna D. Dika E. Boada A. Podlipnik S. Carrera C. (2023). Topographical and chronological analysis of thin cutaneous melanoma’s progressions: a multicentric study. Cancers 15, 3989. 10.3390/CANCERS15153989 37568805 PMC10416930

[B8] Cozzolino C. Buja A. Rugge M. Miatton A. Zorzi M. Vecchiato A. (2023). Machine learning to predict overall short-term mortality in cutaneous melanoma. Discov. Oncol. 14, 13. 10.1007/S12672-023-00622-5 36719475 PMC9889591

[B9] Davis L. E. Shalin S. C. Tackett A. J. (2019). Current state of melanoma diagnosis and treatment. Cancer Biol. and Ther. 20, 1366–1379. 10.1080/15384047.2019.1640032 31366280 PMC6804807

[B10] Ding L. Gosh A. Lee D. J. Emri G. Huss W. J. Bogner P. N. (2022). Prognostic biomarkers of cutaneous melanoma. Photodermatol. Photoimmunol. and Photomed. 38, 418–434. 10.1111/PHPP.12770 34981569

[B11] Dudin O. Mintser O. Kobyliak N. Kaminskyi D. Shabalkov R. Matvieieva A. (2023). Incidence of BRAF mutations in cutaneous melanoma: histopathological and molecular analysis of a Ukrainian population. Melanoma Manag. 10, MMT64. 10.2217/MMT-2023-0005 38221928 PMC10784762

[B12] Ekmekcioglu S. Ellerhorst J. A. Prieto V. G. Johnson M. M. Broemeling L. D. Grimm E. A. (2006). Tumor iNOS predicts poor survival for stage III melanoma patients. Int. J. cancer 119, 861–866. 10.1002/IJC.21767 16557582

[B13] Elder D. E. Gimotty P. A. Guerry D. P. (2005). Cutaneous melanoma: estimating survival and recurrence risk based on histopathologic features. Dermatol. Ther. 18, 369–385. 10.1111/J.1529-8019.2005.00044.X 16297012

[B14] Ewen T. Husain A. Stefanos N. Barrett P. Jones C. Ness T. (2023). Validation of epidermal AMBRA1 and loricrin (AMBLor) as a prognostic biomarker for nonulcerated American Joint Committee on Cancer stage I/II cutaneous melanoma. Br. J. dermatology 190, 549–558. 10.1093/BJD/LJAD459 38006317

[B15] Faries M. B. Thompson J. F. Cochran A. J. Andtbacka R. H. Mozzillo N. Zager J. S. (2017). Completion dissection or observation for sentinel-node metastasis in melanoma. N. Engl. J. Med. 376, 2211–2222. 10.1056/NEJMOA1613210 28591523 PMC5548388

[B16] Faul F. Erdfelder E. Lang A. G. Buchner A. (2007). G*Power 3: a flexible statistical power analysis program for the social, behavioral, and biomedical sciences. Behav. Res. methods 39, 175–191. 10.3758/BF03193146 17695343

[B17] Garbe C. Amaral T. Peris K. Hauschild A. Arenberger P. Basset-Seguin N. (2022). European consensus-based interdisciplinary guideline for melanoma. Part 1: diagnostics: Update 2022. Eur. J. cancer (Oxford, Engl. 1990) 170 (170), 236–255. 10.1016/J.EJCA.2022.03.008 35570085

[B18] Gosman L. M. Țăpoi D. A. Costache M. (2023). Cutaneous melanoma: a review of multifactorial pathogenesis, immunohistochemistry, and emerging biomarkers for early detection and management. Int. J. Mol. Sci. 24, 15881. 10.3390/IJMS242115881 37958863 PMC10650804

[B19] Guitart J. Lowe L. Piepkorn M. Prieto V. G. Rabkin M. S. Ronan S. G. (2002). Histological characteristics of metastasizing thin melanomas: a case-control study of 43 cases. Archives dermatology 138, 603–608. 10.1001/ARCHDERM.138.5.603 12020220

[B20] Helvind N. M. Brinch-Møller Weitemeyer M. Chakera A. H. Hendel H. W. Ellebæk E. Svane I. M. (2023). Stage-specific risk of recurrence and death from melanoma in Denmark, 2008-2021: a national observational cohort study of 25 720 patients with stage ia to IV melanoma. JAMA dermatol. 159, 1213–1222. 10.1001/JAMADERMATOL.2023.3256 37650576 PMC10472263

[B21] Homsi J. Kashani-Sabet M. Messina J. L. Daud A. (2005). Cutaneous melanoma: prognostic factors. Cancer control J. Moffitt Cancer Cent. 12, 223–229. 10.1177/107327480501200403 16258493

[B22] Kaur C. Thomas R. J. Desai N. Green M. A. Lovell D. Powell BWEM (2008). The correlation of regression in primary melanoma with sentinel lymph node status. J. Clin. pathology 61, 297–300. 10.1136/JCP.2007.049411 17675538

[B23] Kunonga T. P. Kenny R. P. W. Astin M. Bryant A. Kontogiannis V. Coughlan D. (2023). Predictive accuracy of risk prediction models for recurrence, metastasis and survival for early-stage cutaneous melanoma: a systematic review. BMJ open 13, e073306. 10.1136/BMJOPEN-2023-073306 PMC10546114 37770261

[B24] Lee S. J. Lim H. J. Choi Y. H. Chang Y. H. Lee W. J. Kim D. W. (2013). The clinical significance of tumor-infiltrating lymphocytes and microscopic satellites in acral melanoma in a Korean population. Ann. dermatology 25, 61–66. 10.5021/AD.2013.25.1.61 PMC3582930 23467045

[B25] Leiter U. Eigentler T. Garbe C. (2014). Follow-up in patients with low-risk cutaneous melanoma: is it worth it? Melanoma Manag. 1, 115–125. 10.2217/MMT.14.22 30190817 PMC6094616

[B26] Liang Z. Pan L. Shi J. Zhang L. (2022). C1QA, C1QB, and GZMB are novel prognostic biomarkers of skin cutaneous melanoma relating tumor microenvironment. Sci. Rep. 12, 20460. 10.1038/S41598-022-24353-9 36443341 PMC9705312

[B27] Lund A. W. (2022). Standing watch: immune activation and failure in melanoma sentinel lymph nodes. Clin. cancer Res. official J. Am. Assoc. Cancer Res. 28, 1996–1998. 10.1158/1078-0432.CCR-22-0214 PMC9106852 35262676

[B28] Mashukov A. Shapochka D. Seleznov O. Kobyliak N. Sulaieva O. Falalyeyeva T. (2021). Histological differentiation impacts the tumor immune microenvironment in gastric carcinoma: relation to the immune cycle. World J. gastroenterology 27, 5259–5271. 10.3748/WJG.V27.I31.5259 PMC8384749 34497449

[B29] McClain S. E. Shada A. L. Barry M. Patterson J. W. Slingluff C. L. (2012). Outcome of sentinel lymph node biopsy and prognostic implications of regression in thin malignant melanoma. Melanoma Res. 22, 302–309. 10.1097/CMR.0B013E328353E673 22610274 PMC4465915

[B30] Morrison S. Han G. Elenwa F. Vetto J. T. Fowler G. Leong S. P. (2022). Is there a relationship between TILs and regression in melanoma? Ann. Surg. Oncol. 29, 2854–2866. 10.1245/S10434-021-11251-Z 35064332

[B31] Naimy S. Bzorek M. Eriksen J. O. Løvendorf M. B. Litman T. Dyring-Andersen B. (2023). LAG3 and TIGIT expression on tumor infiltrating lymphocytes in cutaneous melanoma. Dermatol. Basel, Switz. 240, 156–163. 10.1159/000533932 37952520

[B32] Oláh J. Gyulai R. Korom I. Varga E. Dobozy A. (2003). Tumour regression predicts higher risk of sentinel node involvement in thin cutaneous melanomas. Br. J. dermatology 149, 662–663. 10.1046/J.1365-2133.2003.05502.X 14511013

[B33] Orzan O. A. Șandru A. Jecan C. R. (2015). Controversies in the diagnosis and treatment of early cutaneous melanoma. J. Med. Life 8, 132–141. PMC4392104 25866567

[B34] Peirano D. Donoso F. Vargas S. Hidalgo L. Agüero R. Uribe P. (2023). Patterns of recurrence of cutaneous melanoma: a literature review. Dermatology Pract. and Concept. 13, e2023304. 10.5826/DPC.1304A304 PMC10656145 37992344

[B35] Rashid O. M. Schaum J. C. Wolfe L. G. Brinster N. K. Neifeld J. P. (2011). Prognostic variables and surgical management of foot melanoma: review of a 25-year institutional experience. ISRN Dermatol. 2011, 384729. 10.5402/2011/384729 22363851 PMC3262538

[B36] Rothberg B. E. G. Rimm D. L. (2014). Construction and analysis of multiparameter prognostic models for melanoma outcome. Methods Mol. Biol. Clift. NJ 1102, 227–258. 10.1007/978-1-62703-727-3_13 PMC3912557 24258982

[B37] Salama A. K. S. de Rosa N. Scheri R. P. Pruitt S. K. Herndon J. E. Marcello J. (2013). Hazard-rate analysis and patterns of recurrence in early stage melanoma: moving towards a rationally designed surveillance strategy. PloS one 8, e57665. 10.1371/JOURNAL.PONE.0057665 23516415 PMC3596369

[B38] Serra E. Abarzua-Araya Á. Arance A. Martin-Huertas R. Aya F. Olondo M. L. (2024). Predictive and prognostic factors in melanoma central nervous system metastases-A cohort study. Cancers 16, 2272. 10.3390/CANCERS16122272 38927977 PMC11201698

[B39] Siegel R. L. Miller K. D. Fuchs H. E. Jemal A. (2022). Cancer statistics, 2022. CA a cancer J. Clin. 72, 7–33. 10.3322/CAAC.21708 35020204

[B40] Stakhovskyi O. Kobyliak N. Voylenko O. Stakhovskyi E. Ponomarchuk R. Sulaieva O. (2022). Immune microenvironment of muscular-invasive urothelial carcinoma: the link to tumor immune cycle and prognosis. Cells 11, 1802. 10.3390/CELLS11111802 35681497 PMC9179839

[B41] Stucky C. C. H. Gray R. J. Dueck A. C. Wasif N. Laman S. D. Sekulic A. (2010). Risk factors associated with local and in-transit recurrence of cutaneous melanoma. Am. J. Surg. 200, 770–774. 10.1016/J.AMJSURG.2010.07.025 21146019

[B42] Swetter S. M. Thompson J. A. Albertini M. R. Barker C. A. Baumgartner J. Boland G. (2021). NCCN Guidelines® insights: melanoma: cutaneous, version 2.2021. J. Natl. Compr. Cancer Netw. JNCCN 19, 364–376. 10.6004/JNCCN.2021.0018 33845460

[B43] Tas F. Erturk K. (2017). Histological lymphovascular invasion is associated with nodal involvement, recurrence, and survival in patients with cutaneous malignant melanoma. Int. J. dermatology 56, 166–170. 10.1111/IJD.13405 27778319

[B44] Thompson J. F. Soong S. J. Balch C. M. Gershenwald J. E. Ding S. Coit D. G. (2011). Prognostic significance of mitotic rate in localized primary cutaneous melanoma: an analysis of patients in the multi-institutional American Joint Committee on Cancer melanoma staging database. J. Clin. Oncol. official J. Am. Soc. Clin. Oncol. 29, 2199–2205. 10.1200/JCO.2010.31.5812 PMC3107741 21519009

[B45] Vița O. Jurescu A. Văduva A. Cornea R. Cornianu M. Tăban S. (2023). Invasive cutaneous melanoma: evaluating the prognostic significance of some parameters associated with lymph node metastases. Med. Kaunas. Lith. 59, 1241. 10.3390/MEDICINA59071241 PMC10385614 37512052

[B46] Wan G. Nguyen N. Liu F. DeSimone M. S. Leung B. W. Rajeh A. (2022). Prediction of early-stage melanoma recurrence using clinical and histopathologic features. NPJ Precis. Oncol. 6, 79. 10.1038/S41698-022-00321-4 36316482 PMC9622809

[B47] Wen X. Li D. Zhao J. Li J. Yang T. Ding Y. (2020). Time-varying pattern of recurrence risk for localized melanoma in China. World J. Surg. Oncol. 18, 6. 10.1186/S12957-019-1775-5 31901239 PMC6942369

[B48] Yang B. Xie P. Huai H. Li J. (2023). Comprehensive analysis of necroptotic patterns and associated immune landscapes in individualized treatment of skin cutaneous melanoma. Sci. Rep. 13, 21094. 10.1038/S41598-023-48374-0 38036577 PMC10689831

[B49] Yun S. J. Gimotty P. A. Hwang W. T. Dawson P. Van Belle P. Elder D. E. (2011). High lymphatic vessel density and lymphatic invasion underlie the adverse prognostic effect of radial growth phase regression in melanoma. Am. J. Surg. pathology 35, 235–242. 10.1097/PAS.0B013E3182036CCD PMC3062088 21263244

[B50] Zhang M. Yang L. Wang Y. Zuo Y. Chen D. Guo X. (2023). Comprehensive prediction of immune microenvironment and hot and cold tumor differentiation in cutaneous melanoma based on necroptosis-related lncRNA. Sci. Rep. 13, 7299. 10.1038/S41598-023-34238-0 37147395 PMC10163022

